# In vitro antineoplastic effects of brivaracetam and lacosamide on human glioma cells

**DOI:** 10.1186/s13046-017-0546-9

**Published:** 2017-06-06

**Authors:** Ambra Rizzo, Sara Donzelli, Vita Girgenti, Andrea Sacconi, Chiara Vasco, Andrea Salmaggi, Giovanni Blandino, Marta Maschio, Emilio Ciusani

**Affiliations:** 1 Laboratory of Clinical Pathology and Medical Genetics, Foundation IRCCS Neurological Institute C. Besta, Via Celoria, 11, 20133 Milan, Italy; 2 Oncogenomic and Epigenetic Unit, Regina Elena National Cancer Institute, Via Elio Chianesi, 5300144 Rome, Italy; 3 Neurologia- Stroke Unit, Manzoni Hospital, Via dell’Eremo 9/11, 23900 Lecco, Italy; 4 Center for tumor-related epilepsy, Area of Supporting Care, Regina Elena National Cancer Institute, Via Elio Chianesi 53, 00144 Rome, Italy

**Keywords:** Lacosamide, Brivaracetam, Brain tumor-related epilepsy, Cytotoxicity, Glioma

## Abstract

**Background:**

Epilepsy is a frequent symptom in patients with glioma. Although treatment with antiepileptic drugs is generally effective in controlling seizures, drug-resistant patients are not uncommon. Multidrug resistance proteins (MRPs) and P-gp are over-represented in brain tissue of patients with drug-resistant epilepsy, suggesting their involvement in the clearance of antiepileptic medications. In addition to their anticonvulsant action, some drugs have been documented for cytotoxic effects. Aim of this study was to evaluate possible in vitro cytotoxic effects of two new-generation antiepileptic drugs on a human glioma cell line U87MG.

**Methods:**

Cytotoxicity of brivaracetam and lacosamide was tested on U87MG, SW1783 and T98G by MTS assay. Expression of chemoresistance molecules was evaluated using flow cytometry in U87MG and human umbilical vein endothelial cells (HUVECs). To investigate the putative anti-proliferative effect, apoptosis assay, microRNA expression profile and study of cell cycle were performed.

**Results:**

Brivaracetam and lacosamide showed a dose-dependent cytotoxic and anti-migratory effects. Cytotoxicity was not related to apoptosis. The exposure of glioma cells to brivaracetam and lacosamide resulted in the modulation of several microRNAs; particularly, the effect of miR-195-5p modulation seemed to affect cell cycle, while miR-107 seemed to be implicated in the inhibition of cells migration. Moreover, brivaracetam and lacosamide treatment did not modulate the expression of chemoresistance-related molecules MRPs1-3-5, GSTπ, P-gp on U87MG and HUVECs.

**Conclusion:**

Based on antineoplastic effect of brivaracetam and lacosamide on glioma cells, we assume that patients with glioma could benefit by the treatment with these two molecules, in addition to standard therapeutic options.

**Electronic supplementary material:**

The online version of this article (doi:10.1186/s13046-017-0546-9) contains supplementary material, which is available to authorized users.

## Background

Brain tumors are among the most disabling and resource-consuming diseases in neurology [1–4]. A common clinical manifestation of brain tumors is brain tumor-related epilepsy; in younger patients, with a long life expectancy, epilepsy is often the major problem, whereas in patients with high grade gliomas, seizures are part of a more complex scenario involving cognitive deterioration and motor/sensory deficits [5–7].

The management of brain tumor-related epilepsy must keep in consideration issues pertaining to both epileptology and neuro-oncology. Side effects of anti-epileptic drugs (AED) have in fact a relevant role; indeed, enzyme-inducing anticonvulsant drugs (EIAED) may interfere with the metabolism of chemotherapeutic agents [8, 9]. Moreover, antiepileptic drugs per se may display an activity on brain cancer cells, as suggested by in vitro experiments [10].

In 2009 Jaeckle had analysed prospectively the EIAED use in correlation with the outcome in patients with newly diagnosed glioblastoma (GBM). They found that overall survival (OS) and progression-free survival (PFS) were significantly longer in patients receiving EIAED compared to patients receiving non-EIAED [11].

On the other hand, in 2011 Weller et al. examined the impact of the interaction between AED use and chemo-radiotherapy on survival in patients with newly diagnosed GBM. The OS of patients who were receiving AED at baseline versus not-receiving any AED was similar. However, patients receiving valproic acid (VPA) alone appeared to derive more survival benefit from chemo-radiotherapy (HR 0.39, 95% CI: 0.24–0.63) than patients receiving EIAED (HR 0.69, 95% CI: 0.53–0.90) or patients not receiving any AED (HR 0.67, 95% CI: 0.49–0.93) [12].

In this context, particular attention has been devoted to two non-EIAED: VPA, known for a long time as a histone deacetylase inhibitor [13] and levetiracetam (LEV).

In 2013, Guthrie investigated the role of VPA as an antitumor agent in the management of patients with GBM. The results showed that patients treated with AED had a significantly longer survival than those who were not. Moreover, patients receiving VPA had a significantly longer survival than both those who did not receive AED and those who received other AED [14].

Kerkhof studied the effect of VPA on survival of patients with newly diagnosed GBM. The group using VPA and temozolomide (TMZ) during at least 3 months had a significantly longer median survival compared with what observed in the group not using VPA or using another AED (69 vs 61 weeks) [15].

As far as LEV is concerned, in 2010 Bobustuc hypothesized that AED may modulate O-6 methylguanine-DNA methyltransferase (MGMT), a DNA repair protein that has an important role in tumor cell resistance to alkylating agents, and LEV was reported as the most potent MGMT inhibitor among several AED [10].

Recently, Kim analysed the benefit of LEV compared with other AED as a chemosensitizer to TMZ for patients with GBM [16]. The median PFS and OS for patients who received LEV in combination with TMZ were significantly longer than those for patients who did not receive LEV (6.7 vs 9.4 months and 16.7 vs 25.7 months respectively).

The possible actions of antiepileptic drugs on brain cancer cells include activity on cell proliferation, apoptosis and migration [17]. A number of intracellular pathways are involved in these activities, among which microRNA (miRNA) are gaining increasing attention. Many data suggest that miRNA are key components of a wide range of biological processes [18–20] and in a recently published study, in which miRNAs expression profiling was analyzed in a cohort of patients affected by low grade-gliomas, miR-196b has been identified as a predictive marker of seizures occurrence [21].

Among drugs recently introduced in the management of epilepsy, both lacosamide (LCM) and brivaracetam (BRV) are devoid of enzyme-inducing activity on the cytochrome system, being good candidates for introduction in the management of brain tumor epilepsy. Lacosamide has an inhibitory activity on histone deacetylase [22], making it worthwhile investigating its in vitro effects on brain cancer, while brivaracetam, a parent compound of levetiracetam, might share with it the same biological effects and both have been recently described to have high brain permeability [23, 24].

In the present study, we investigated in vitro on a human glioma cell line, the effects of brivaracetam and lacosamide on biological parameters involved in tumor growth, resistance to therapies and invasiveness.

## Methods

### Cell cultures

The human glioma cell lines U87MG, SW1483 and T98G and human fibroblasts were purchased from ATCC (LGC Standards S.r.l.). Cells were cultured in Dulbecco’s Modified Eagle’s Medium (DMEM, Gibco) supplemented with 10% foetal bovine serum (FBS, Gibco) and 1% penicillin/streptomycin (Gibco).

Human umbilical vein endothelial cells (HUVECs) primary cultures were isolated from healthy donors [25] and used in experiments up to the tenth passage. Purity of cell cultures was higher than 95% as assessed by flow cytometry after CD31 staining (polyclonal phycoerythrin-conjugated goat anti-human CD31, BD Bioscience). HUVECs were cultured in complete Endothelial Basal Medium (Lonza) supplemented with growth factors and antibiotics (EGM Single Quots, Lonza).

### Drugs

BRV and LCM were kindly provided by UCB Pharma. Drugs were dissolved in distilled water at a concentration of 100 mM for BRV and 50 mM for LCM and successively diluted in complete medium to the necessary experimental concentrations (BRV: 100-200-400-600-800-1000-1200-1800-2000 μM; LCM: 100-200-400-600-800-1000-1200-1600-2000-2400 μM).

### Cytotoxicity

Cytotoxicity of BRV and LCM was studied by cell proliferation assay following the manufacturer’s protocol (MTS assay, Molecular Probes).

Inhibitory concentrations (IC) were calculated from the regression line that associates percentage of inhibition and drug concentrations as follows: 100-(100 x average n.cells x C/n. cell basal level). Where C = drug concentration [range 0–2500 μM].

### Apoptosis

Evaluation of apoptotic cells was performed in treated cells (IC20 BRV or IC20 LCM at 24–48–72 h) and in untreated cells (control) using Annexin V binding assay (Immunostep) for flow citometry (FacsVantage SE, Becton Dickinson) following the manufacturer’s protocol.

### Chemoresistance

Treated and untreated cells were fixed and permeabilized using Cytofix/CytopermTM Fixation/Permeabilization Kit (BD Biosciences) for the evaluation of the expression of chemoresistance-associated molecules. Cells were then stained with the following antibodies: mouse-antihuman MRP1 (Monosan), MRP3 (Abcam), GSTπ (LSbio), P-gP (Chemicon) and rat–anti-human MRP5 (Kamiya) primary antibodies for 1 h at 4 °C and subsequently 30 min at 4 °C with a secondary conjugated antibody for flow cytometry: goat-anti-mouse fluorescein isothiocyanate-conjugated antibody (BD Biosciences) for MRP1, MRP3, GSTπ and P-gP and goat anti rat-biotin streptavidin phycoerythrin conjugated antibody for MRP5. Cells were analyzed by flow-cytometry.

### Cell cycle

Cells (8 · 10^5) were plated in triplicate and cultured for 24 h. LCM 300 μM (IC20) and BRV 400 μM (IC20) were then added for 72 h. At each time-point cells were harvested and fixed in ethanol 80% at 4 °C for 30 min, washed and stained with PI 50 μg/ml in PBS overnight at 4 °C. DNA content was evaluated using flow-cytometry.

### MiRNA expression

Total RNA was extracted using the TRIZOL (Gibco). The concentration and purity of total RNA were assessed using a Nanodrop TM 1000 (Nanodrop Technologies). Total RNA (100 ng) was labelled and hybridized to Human miRNA Microarray V.19 (Agilent). Scanning and image analysis were performed using the Agilent DNA Microarray Scanner (P/N G2565BA) equipped with extended dynamic range (XDR) software according to the Agilent miRNA Microarray System with miRNA Complete Labeling and Hyb Kit protocol manual. Feature Extraction Software (Version 10.5) was used for data extraction from raw microarray image files using the miRNA_105_Dec08 FE protocol.

### Formaldehyde cross-linking and chromatin immunoprecipitation

Formaldehyde cross-linking and chromatin immunoprecipitations were performed as previously described. The chromatin solution was immunoprecipitated with anti-H4K8ac (Cell Signaling), anti-H3K9me3 (Cell Signaling).

### Bioinformatics analysis

Array analysis was performed using Matlab (The MathWorks Inc.). Signals were extracted using Agilent Feature Extraction, quantile normalized and log2-trasformed. Paired and unpaired T-test were applied to evaluate significantly deregulated miRNAs. For signature selection we considered as significant *p* values less than 0.01. A False Discovery Rate procedure for multiple comparisons was also included in the analysis. Hierarchical Clustering and Principal Component Analysis were used to evaluate the efficacy of the selected signature.

Target prediction was assessed by using several prediction software included in the web server tool MirWalk2.0 (http://zmf.umm.uni-heidelberg.de/apps/zmf/mirwalk2/). Prediction was considered reliable if confirmed by at least three different software. Predicted targets were used for pathway analysis.

### qRT-PCR analysis

10 ng of RNA was reverse-transcribed using the TaqMan microRNA Reverse Transcription Kit (Applied Biosystem) and Real time-PCR of miR expression was carried out using ABI Prism 7000 Sequence Detection System (Applied Biosystems). The PCR Reactions were initiated with a 10 min incubation at 95 °C followed by 40 cycles of 95 °C for 15 s and 60 °C for 60 s. RTq-PCR quantification of miRNA expression was performed using TaqMan MicroRNA® Assays (Applied Biosystems) according to the manufacturer’s protocol. RNU48 was used as endogenous control to normalize microRNA expression. All reactions were performed in duplicate.

### Transfection

For mature miR-195-5p or miR-107 expression, we used Pre-miRNA Precursor-Negative Control (Ambion) and Pre-miRNA195-5p (Ambion) or Pre-miRNA107 at final concentration of 5nM. For miR-195-5p and miR-107 depletion we used miRCURY LNA microRNA inhibitor control (Exiqon) and hsa-miR-195-5p miRCURY LNA (Exiqon) or hsa-miR-107 miRCURY LNA (Exiqon) at final concentration of 10nM. U87MG cells were transfected using Lipofectamine RNAiMAX (Invitrogen) according to the manufacturer’s instructions. For miRNAs depletion experiments, after 48 h of transfection cells were treated with IC20 BRV or IC20 LCM for 48 h.

### Immunoblotting analysis

Cells were lysed in buffer consisting of 50 mM Tris-HCl pH 8, with 1% NP-40 (Igepal AC-630) 150 mM NaCl, 5 mM EDTA and fresh protease inhibitors. Protein concentrations were determined by colorimetric assay (Bio-Rad). Western blotting was performed using the following primary antibodies: mouse monoclonal anti-Tubulin (Santa Cruz Biotechnology), mouse monoclonal anti-Gapdh (Santa Cruz Biotechnology), rabbit polyclonal anti-p21 (Santa Cruz Biotechnology), rabbit polyclonal anti-Cyclin A (Santa Cruz Biotechnology), mouse monoclonal anti-Cyclin E (Santa Cruz Biotechnology), rabbit monoclonal anti-EGFR (Cell Signaling Tecnology, C74B9), rabbit polyclonal anti-N-Cadherin (Abcam). Secondary antibodies used were goat anti-mouse and goat anti-rabbit conjugated to horseradish peroxidase (Santa Cruz Biotechnology).

### Cell proliferation assay

U87MG cells (6 × 104) were transfected in triplicated as indicated. Cells were collected and counted at 0–24–48–72 h after transfection.

### Migration assay

Migration was measured using a 24-well plate with a non-coated 8-mm pore size filter in the insert chamber (BD Falcon). Cells were transfected with Pre-miRNA Precursor-Negative Control or the Pre-miRNA107, or the Pre-miRNA195-5p (Ambion), or treated with BRV or LCM at IC20. After 48 h from transfection or treatments, cells were resuspended in DMEM medium without FBS and seeded into the insert chamber. Cells were allowed to migrate for 12 h into the bottom chamber containing 0.7 ml DMEM medium containing 10% FBS in a humidified incubator at 37 °C in 5% CO2. Migrated cells that had attached to the outside of the filter were visualized by staining with DAPI and counted.

### Statistical analysis

Statistical analyses were performed by Pearson correlation coefficient for cytotoxicity assay and by Student-t test for apoptosis, molecular analysis and cell cycle. Unless differently specified, level of significance was set at *p* < 0.05.

## Results

### Brivaracetam and lacosamide exert cytotoxic effect on glioma cells and inhibit cell migration

**Fig. 1:**
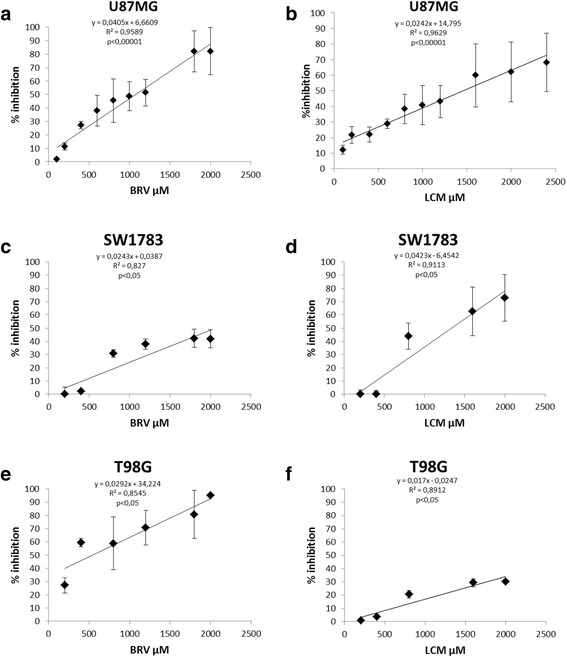
*Brivaracetam and lacosamide treatments exert a cytotoxic effect on U87MG, SW1783 and T98G glioma cells exposed to increasing drugs concentration.* Graphs show the cytotoxic effect of BRV and LCM on U87MG cell line (**a**-**b**), Pearson correlation index *p* <0.00001 for both), SW1783 (**c**-**d**), Pearson correlation index *p* <0.05 for both) and T98G (**e**-**f**), Pearson correlation index *p* <0.05 for both). Data are expressed as % of inhibition calculated with the formula: 100-(100 x mean cell number x C/n.cell basal level) where C = drug concentration [range 0–2500 μM]. Data refer to at least three independent experiments, error bars represent the SD

In our experimental conditions, BRV and LCM displayed a dose-dependent cytotoxic effect in all glioma cell lines tested (Fig. 1) while no cytotoxic effect was detected in normal human fibroblasts (Additional file 1: Figure S1). A more detailed analysis in U87 revealed that the IC20 (i.e. 20% growth inhibiting concentration) were 400 μM for BRV and 300 μM for LCM. In T98G and SW1783 cell lines, IC20 were respectively 200 μM and 821 μM for BRV and 1178 μM and 625 μM for LCM.

No statistically significant effect of BRV or LCM was observed on apoptosis in U87MG. Even if a trend to increased apoptosis was observed 72 h after treatment with both drugs, this affects less than 4% of the cells (Additional file 2: Figure S2a). Similarly, HUVECs did not display a statistically significant increase in apoptosis after in vitro exposure to the two drugs (Additional file 2: Figure S2b).

**Fig. 2:**
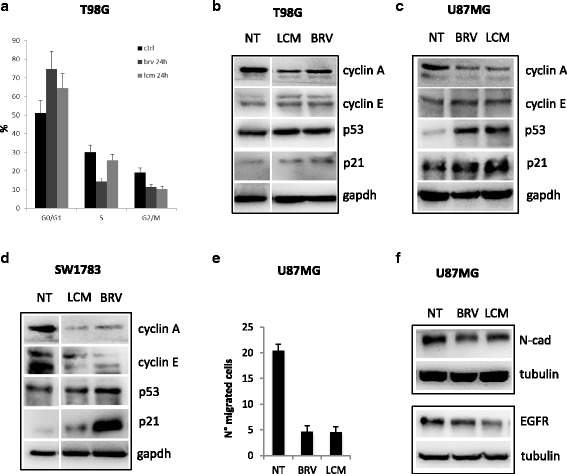
*Brivaracetam and lacosamide treatments exerted an anti-proliferative effect and a reduction in migration ability of glioma cells*. **a** Distribution of T98G cells in the different phases of the cell cycle upon 24 h BRV or LCM treatments (IC20). Data are expressed as percentage of cell in a specific phase (G0/G1, S, G2/M) and refers to at least four independent experiments. Statistical evaluation was performed by the student’s t-test. Histogram bars represent mean ± standard deviation of at least three independent replicates. **b**-**c**-**d** Western-blot analysis of cyclin A, cyclin E, p53 and p21 upon treatments with BRV or LCM at IC20 in T98G **a** U87MG **b** SW1783 **c** cells. **e** Transwell migration assay in U87MG cells upon BRV or LCM treatments. (IC20). **f** Western-blot analysis of EGFR and N-cadherin proteins expression levels in U87MG cells upon BRV or LCM treatments (IC20)

Upon BRV or LCM treatments (IC20) in T98G cells, we observed a specific arrest in G1 phase of cell cycle (Fig. 2a and Additional file 3: Figure S3 a-b). This effect on cell cycle was also confirmed by an increase in p21 protein expression levels, by a reduction in cyclin A protein levels and a concomitant accumulation of cyclin E protein observed in T98G, U87MG and SW1783 glioma cells upon BRV or LCM (IC20) treatments (Fig. 2b-d). To dipper investigate BRV and LCM effect on cell cycle, we also checked p53 protein levels upon BRV or LCM (IC20) observing an increase in its levels mainly in U87MG cells (p53 wild-type) and less evident in T98G and SW1783 cells (mutant p53) (Fig. 2b-d). This result suggested a specific effect of BRV and LCM on p53 protein depending on its mutational status.

Moreover, both treatments significantly impinged on U87MG cells migration (Fig. 2e). This effect was also confirmed by the reduction in N-cadherin and EGFR proteins expression levels in the same experimental conditions (Fig. 2f).

### Brivaracetam and lacosamide treatments do not impinge on multidrug resistance proteins expression

Since regulation of multidrug resistance proteins expression might directly increase chemoresistance of glioma cells or decrease the permeability of the blood brain barrier to chemotherapic drugs, the expression of multidrug resistance proteins MRP1, MRP3, MRP5, PgP and GSTπ was evaluated on U87MG cells and HUVEC after exposure to BRV and LCM IC20. No statistically significant differences in protein expression were observed after drug treatments compared to untreated cells (Additional file 4: Figure S4).

### Brivaracetam and lacosamide treatments modulate miRNAs expression in glioma cells

**Fig. 3:**
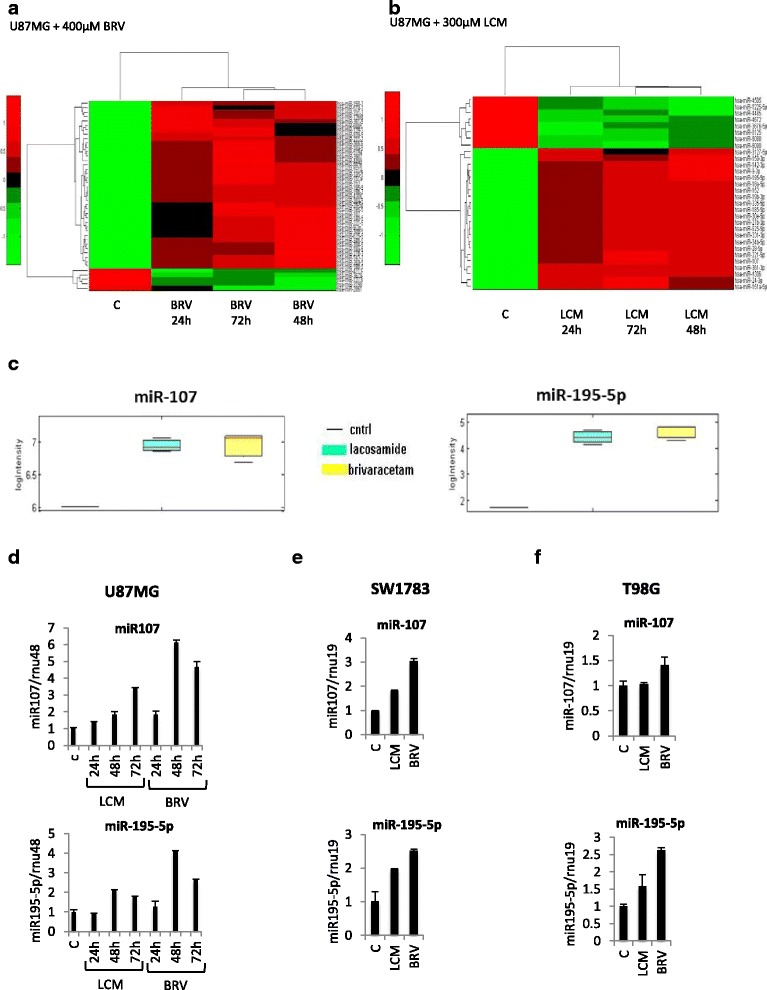
*Brivaracetam and lacosamide treatments modulate microRNAs expression in U87MG glioma cells.* **a** Heat map of the identified signature of 37 microRNAs differentially expressed in U87MG cells treated with BRV at IC20 at the indicated time points. **b** Heat map of the identified signature of 30 microRNAs differentially expressed in U87MG cells treated with LCM at IC20 at the indicated time points. **c** Supervised statistical test of the significance level of the difference between signal distributions of miR-107 and miR-195-5p in U87MG cells treated with LCM or BRV versus the control (untreated cells). **d**-**e**-**f** qRT-PCR validation of miR-107 and miR-195-5p in U87MG, SW1783 and T98G upon IC20 LCM or BRV treatments at the indicated time points

To investigate if cytotoxic effect of BRV and LCM was mediated by miRNAs, we performed a miRNAs expression profile. We profiled the expression of 2000 human miRNAs in U87MG treated with BRV or LCM IC20 at the indicated time points (Fig. 3a-b). This kind of analysis identified a signature of 43 and 30 modulated miRNAs in U87MG treated with BRV and LCM respectively, 20 of which were shared by the two treatments (Fig. 3a-b, Additional file 5: Figure S5 and Additional file 6: Figure S6).

By performing pathway analysis of the putative target of both miRNAs signatures, we observed an almost complete overlapping between BRV and LCM treatments, possibly indicating a similar mechanism of action of the two AEDs (Table 1).

**Table 1:** Predicted pathwhays targeted by miRNAs modulated by Brivaracetam and Lacosamide

KEGG pathway	*p*-value	#genes	#miRNAs
BRIVARACETAM			
Proteoglycans in cancer	6.85E-09	130	35
Fatty acid biosynthesis	7.19E-08	8	11
TGF-beta signaling pathway	1.74E-07	55	32
AMPK signaling pathway	5.90E-07	84	36
Hippo signaling pathway	1.68E-06	93	33
Glutamatergic synapse	7.59E-06	72	35
Long-term depression	1.70E-05	42	32
Adrenergic signaling in cardiomyocytes	1.70E-05	92	37
Pathways in cancer	1.78E-05	219	38
Axon guidance	3.75E-05	79	34
LACOSAMIDE			
Proteoglycans in cancer	5.142E-11	120	22
Fatty acid biosynthesis	5.755E-10	8	10
Hippo signaling pathway	5.755E-10	92	24
Axon guidance	2.903E-07	77	20
Long-term depression	4.614E-07	43	21
TGF-beta signaling pathway	1.372E-06	49	20
Fatty acid metabolism	1.636E-06	23	17
Adrenergic signaling in cardiomyocytes	1.771E-06	86	24
Signaling pathways regulating pluripotency of stem cells	3.548E-06	82	22
Glutamatergic synapse	3.548E-06	66	23

We focused our attention on two miRNAs whose expression was induced by both treatments compared to the control (untreated cells) and whose role in glioma tumorigenesis was partly characterized: miR-107 and miR-195-5p (Fig. 3c) [26–31]. To confirm these results, we analysed the expression levels of miR-107 and miR-195-5p by qRT-PCR (Fig. 3d). These analyses confirmed the up-regulation of both miRNAs upon BRV and LCM IC20 treatments at the indicated time points. Although at a minor extent, the increase in miR-195-5p expression was detected also in SW1783 and in T98G cell line upon treatments with both drugs, while in the latter cell line miR-107 was not inducible by LCM (Fig. 3e-f).

### Brivaracetam and lacosamide treatments induce miR-107 and miR-195-5p expression in glioma cells by determining epigenetic modification on their regulatory regions

**Fig. 4:**
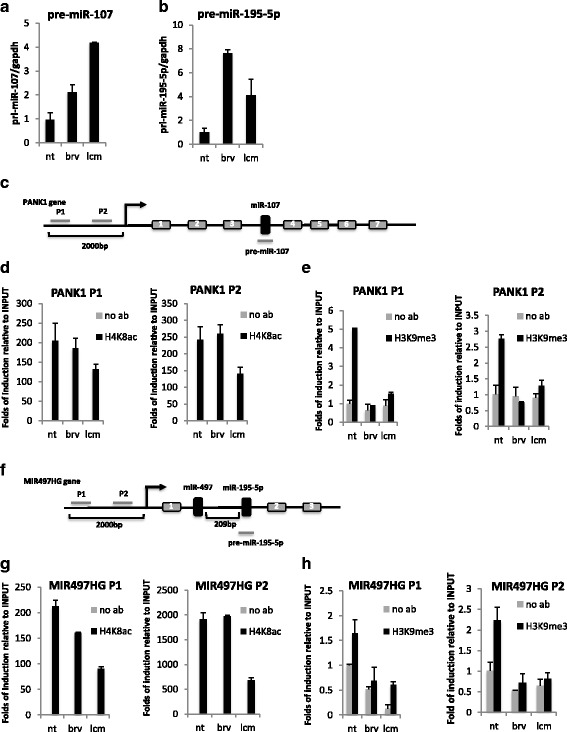
*Brivaracetam and lacosamide treatments induce epigenetic modification on miR-107 and miR-195 regulatory regions.* **a**-**b** qRT-PCR of miR-107 **a** and miR-195-5p **b** precursors (pre-miRNAs) in U87MG cells upon IC20 LCM or BRV treatments. **c** Schematic representation of miR-107 gene locus: two different regions of host gene (PANK1) promoter have been analyzed in ChIP assays (P1 and P2). **d**-**e** ChIP analysis of acetylated histone H4 and methylated histone H3 occupancy on miR-107 regulatory regions in U87MG cells upon treatment with BRV or LCM at IC20. **f** Schematic representation of miR-195-5p gene locus: two different regions of host gene (MIR497HG) promoter have been analyzed in ChIP assays (P1 and P2). **g**-**h** ChIP analysis of acetylated histone H4 and methylated histone H3 occupancy on miR-195-5p regulatory regions in U87MG cells upon treatment with BRV or LCM at IC20

To evaluate if BRV- and LCM-mediated modulation of miR-107 and miR-195-5p expression occurred at the transcriptional level, we analysed the levels of their precursors (pre-miRNAs) upon BRV and LCM treatments in U87MG cells. As shown in Fig. 4a-b, both two AEDs increase pre-miRNAs levels, suggesting a transcriptional regulation of miR-107 and miR-195-5p.

To deeper investigate the molecular mechanism involved in this regulation, we evaluate the ability of BRV and LCM to induce epigenetic modifications on the regulatory regions of miR-107 and miR-195-5p genes. In particular, for miR-107, which is localized in the third intron of PANK1 gene, by analysing two regions of PANK1 promoter, we didn’t observed a significant modification in the acetylation status of histone H4, that resulted highly acetylated in the control cells (untreated cells), while we observed a reduction in the methylation status of histone H3 in (Fig. 4c-e). Similar results were obtained for miR-195-5p, localized in the first intron of MIR495HG gene, by analysing two regions of MIR495HG promoter (Fig. 4f-h).

Altogether these findings indicated a BRV- and LCM-mediated chromatin remodeling effect on miR-107 and miR-195 genes, in particular a reduction in the methylation status, determining miRNAs expression.

### MiR-195-5p inhibits U87MG cells proliferation

**Fig. 5:**
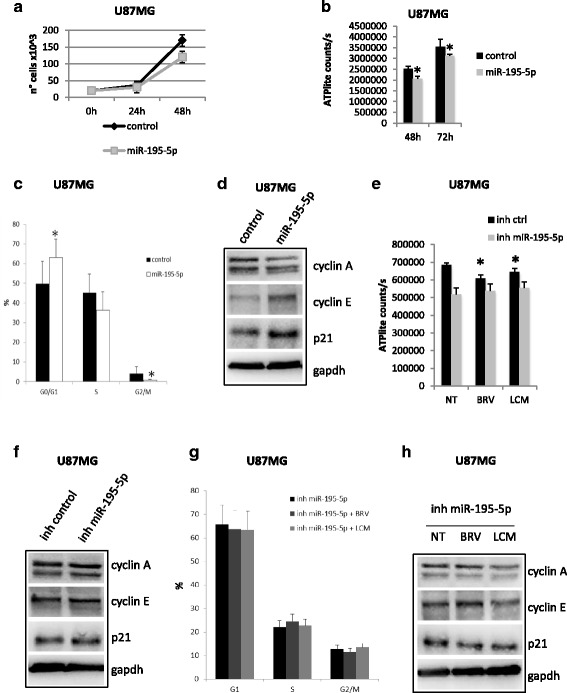
*Brivaracetam and lacosamide treatments exert their anti-proliferative effect in part trough miR-195-5p.* **a**-**b** Proliferation assay (**a**) and viability assay **b** in U87MG cells transfected with miR-195-5p mimic or control. Cells were collected and counted at the indicated time points. **c** Distribution of U87MG cells in the different phases of the cell cycle. Data are expressed as percentage of cell in a specific phase (G0/G1, S, G2/M) and refers to at least four independent experiments. Statistical evaluation was performed by the student’s t-test. Histogram bars represent mean ± standard deviation of at least three independent replicates. **d** Western-blot analysis of cyclin A, cyclin E and p21 protein expression levels in U87MG cells upon miR-195-5p over-expression. **e** Viability assay in U87MG cells transfected with miR-195-5p inhibitor or control and treated with BRV or LCM at IC20. **f** Western-blot analysis of cyclin A, cyclin E and p21 protein expression levels in U87MG cells upon miR-195-5p depletion with miR-195-5p inhibitor (inh miR-195-5p). **g** Distribution of U87MG cells in the different phases of the cell cycle upon miR-195 depletion and treatments wit BRV or LCM at IC20. Data are expressed as percentage of cell in a specific phase (G0/G1, S, G2/M) and refers to at least four independent experiments. Statistical evaluation was performed by the student’s t-test. Histogram bars represent mean ± standard deviation of at least three independent replicates. **h** Western-blot analysis of cyclin A, cyclin E and p21 protein expression levels in U87MG cells upon miR-195-5p depletion with miR-195-5p inhibitor (inh miR-195-5p) and treatments wit BRV or LCM at IC20. (* = *p*val < 0.05)

To test if the cytotoxic effect of BRV and LCM was in part mediated by the induction of miR-195-5p or miR-107, we ectopically expressed miR-195-5p or miR-107 in U87MG and we evaluated cell proliferation rate. MiR-195-5p expression determined a significant reduction in U87MG growth and viability (Fig. 5a-b). On the other hand, miR-107 overexpression didn’t determine any change (Additional file 7: Figure S7a-b).

Cell cycle analysis was also performed in U87MG cells ectopically expressing miR-195-5p (Fig. 5c and Additional file 7: Figure S7c). Overexpression of miR-195-5p induced a significant increase in the percentage of cells in G0/G1 phase of the cell cycle after 48 h from transfection and a concomitant decrease in G2/M (Fig. 5c and Additional file 7: Figure S7c). These results are in line with the observed increase in p21 and cyclin E protein levels and decrease in cyclin A levels upon miR-195-5p over-expression or upon treatment with BRV or LCM (Figs. 5d and 2b-d). The anti-proliferative effect exerted by miR-195-5p was confirmed by cell morphology which presented the typical features of growth-arrested cells (Additional file 7: Figure S7d).

On the contrary, mir-195-5p depletion had no effect on U87MG cells viability and did not determine any change in cyclin A, cyclin E and p21 protein levels (Fig. 5e-f Additional file 7: Figure S7e). Moreover, as shown in Fig. 5g-h, mir-195-5p silencing in U87MG cells abolished the anti-proliferative effects of BRV or LCM treatments. In particular miR-195-5p depletion blocked the BRV- or LCM- induced arrest in G1 phase of cell cycle and prevented cyclin A protein reduction and accumulation of cyclin E and p21 proteins (Fig. 5g-h).

### MiR-107 promotes U87MG cells migration

**Fig. 6:**
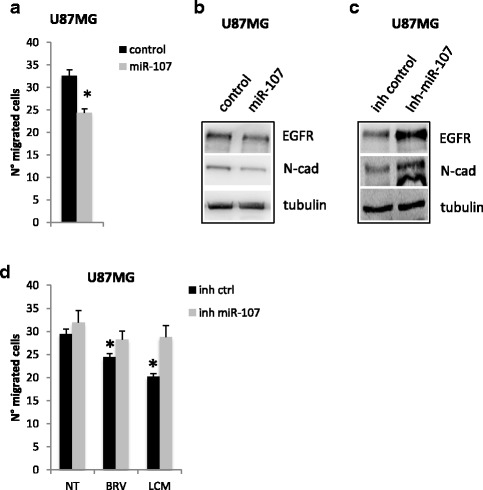
*Brivaracetam and lacosamide treatments inhibit glioma cells migration in part trough miR-107.* **a** Transwell migration assay in U87MG cells upon miR-107 exogenous expression (* = *p*val < 0.05). **b**-**c** Western-blot analysis of EGFR and N-cadherin proteins expression levels in U87MG cells upon miR-107 over-expression with miR-107 mimic (miR-107) (**b**) or depletion with miR-107 inhibitor (inh miR-107) (**c**). **d** Transwell migration assay in U87MG cells upon miR-107 depletion with miR-107 inhibitor (inh miR-107)

BRV and LCM treatments in U87MG also determined a significant reduction in cell migration (Fig. 2e). We evaluated if this effect was mediated by miR-195-5p or miR-107 modulation. MiR-107 significantly impinged on cell migration, while miR-195-5p had no effect (Fig. 6a and Additional file 8: Figure S8a). The inhibitory effect on cell migration was also verifiable in the levels of EGFR and N-Cadherin proteins that were strongly reduced by miR-107 over-expressing cells (Fig. 6b) or by BRV and LCM treatments (Fig. 2c). On the contrary, miR-107 depletion improve migratory ability of U87MG cells as indicated by the increase of EGFR and N-cadherin protein levels (Fig. 6c). Moreover, miR-107 depletion prevented BRV- and LCM-mediated inhibition of cell migration (Fig. 6e and Additional file 8: Figure S8b).

## Discussion

Epilepsy is a frequent complication in patients with brain tumors, therefore AED are widely used for seizure control in addition to surgery, chemotherapy and radiotherapy. For this reason a possible intrinsic antineoplastic effect of these drugs would be useful. In addition to the anticonvulsant mechanism, some AED have been previously described to exert a cytotoxic effect that, added to the effects of conventional chemo-radiotherapy, possibly impacts also on the survival of these patients [10, 16, 17]. Our results show that BRV and LCM in vitro exert a dose-dependent cytotoxic effect on various glioma cell lines and this effect was concomitant with the modulation of a number of miRNAs.

Although we could not completely clarify the mechanism of action, our data suggest that, even at low doses (IC20), the two drugs exert a role in blocking cell cycle progression of glioma cells possibly trough up-regulation of miR-195-5p. Indeed, in our experimental conditions, these two drugs seem to impinge on G1/S phase transition of cell cycle, as demonstrated by reduction in cyclin A protein levels and increase in p21 and cyclin E protein levels after treatments.

The tumor-suppressor activity of miR-195-5p in glioma cells and other tumor models has been previously characterized [29–31]. Zhang et al. reported that overexpression of miR-195-5p was able to induce the arrest of cell cycle progression in G1/S transition in U87 [31] and similar results have been described by Hui et al. in other cellular models of human glioma [29]. An increase in p21 expression has been also previously reported to be associated to cell cycle arrest in lung, colorectal, thyroid and kidney cell lines [32–34]. In agreement with these data, our results confirm the role of miR-195-5p in the suppression of cell proliferation in human glioma cells.

Other AED have been previously reported to interfere with cell cycle in cancer cells [35–37]. Bobustuc and colleagues reported that levetiracetam inhibited in vitro human glioma cell proliferation through p53-mediated MGMT inhibition, thus increasing glioma cell sensitivity to temozolomide [10]. Moreover, in a recent clinical study, Kim and colleagues showed that patients with glioblastoma receiving temozolomide-based chemotherapy and levetiracetam for seizure control, experienced a significant survival benefits [16]. Our in vitro data suggest that some characteristics of the parent drug levetiracetam might be shared by BRV, however further study is needed to verify the effect of BRV on MGMT expression.

Moreover, our results show that BRV and LEV may modulate other miRNAs. Over-expression of miR-107 has been also demonstrated to inhibit glioma cell growth increasing apoptosis [28] and to inhibit cell migration/invasion [26, 27]. In our experimental conditions after treatment with LCM or BRV, the percentage of apoptotic cells was barely detectable therefore too low to be responsible of the observed cytotoxicity. On the other hand, our data confirm that overexpression of miR-107 reduces the ability of glioma cells to migrate in an in vitro assay.

Exposure to BRV and LCM seems to have no impact on chemoresistance. Indeed ATP-dependent drug efflux transporters or multidrug resistance proteins have been localized in different tissues including the blood brain barrier and they affect absorption, distribution and excretion of different drugs [38–41]. Although preliminary, our results on endothelial and glioma cells suggest that the administration of these two drugs would not significantly modify glioma cells chemoresistance or the availability of chemotherapic drugs at the blood/tumor interface.

The meaning of the decreased expression of N-cadherin and EGFR following miR-107 overexpression deserves further investigation.

In U87 cells the decrease in N-cadherin protein levels correlates with a reduced migratory ability of the cells upon ectopic expression of miR-107 as upon treatment with BRV or LCM. The role of N-cadherin in the migration of mesenchymal cells is not completely defined. Recently, Guo et al. observed a reduction in the expression of mesenchymal related protein, such as N-cadherin, that determined a mesenchymal-to-epithelial transition of U87 and a consequent inhibition of cell migration [42]. This is in line with our observations.

Amplified expression of EGFR and of its mutated variant v3, has been extensively studied as a possible target for anti-tumor therapy, although clinical trials focused on this treatment approach have so far yielded unsatisfactory results [43]. A positive correlation between EGFR expression and migration ability has been previously reported in glioma cell lines and in neuronal stem cells therefore our results would further support the role of EGFR in cell motility [44, 45].

## Conclusions

Even if at concentration higher than those recommended for epilepsy control, our study provides evidence for possible effects of two novel anti-epileptic drugs, both devoid of enzyme-inducing activity, on glioblastoma cell proliferation/migration, at least partly mediated by enhanced expression of 2 miRNAs.

Further studies are needed to better delineate the multiple biological effects of these drugs in glioma and their possible impact on clinical management and outcome in brain tumor-related epilepsy.

## Additional files

Additional file 1: Figure S1. Brivaracetam and lacosamide treatments displayed no cytotoxic effect on normal human fibroblast exposed to increasing drugs concentration. Data refers to at least three independent experiments and are expressed as cell number ± SD. (PPTX 106 kb)

Additional file 2: Figure S2. a-b) Apoptosis analysis studied on the cell line U87MG (a) and on HUVECs (b) after treatment with BRV (IC20, grey histogram) and LCM (IC20, empty histogram) at different time points 24, 48 and 72 h. Data are expressed as difference in the % of apoptotic cells between treated and untreated cells (Δ apoptotic inhibition). Data refer to at least three independent experiments, error bars represent the SD. (PPTX 72 kb)

Additional file 3: Figure S3. *Brivaracetam and lacosamide treatments induces accumulation of cells in G0/G1.* a-b) Distribution of T98G cells in the different phases of the cell cycle upon 48 h (a) or 72 h (b) BRV or LCM treatments (IC20). Data are expressed as percentage of cell in a specific phase (G0/G1, S, G2/M) and refers to at least four independent experiments. Statistical evaluation was performed by the student’s t-test. Histogram bars represent mean ± standard deviation of at least three independent replicates. (PPTX 65 kb)

Additional file 4: Figure S4. *Brivaracetam and lacosamide treatments have no impact on chemoresistence induction*. Drug resistance molecules expression and modulation in U87MG cell line as detected by flow cytometry. In vitro cultured cells were treated with an IC20 concentration of BRV (grey histogram) or LCM (empty histogram) for 72 h, harvested and labelled with the specific antibodies (see methods). Data are expressed as fold increase/decrease (means ± SD) of treated cells compared to basal expression (averages of mean fluorescence intensity of treated cells/averages of mean fluorescence intensity of untreated cells). (PPTX 196 kb)

Additional file 5: Figure S5. *miRNAs modulated in U87MG cells upon LCM treatment.* Differentiating miRNAs are listed with their *p*-values obtained by paired t-test (*p*val). In the table are also indicated false discovery rate values (FDR), and folds of deregulation expressed in logarithmic scale (log2 fold). (PPTX 63 kb)

Additional file 6: Figure S6. *miRNAs modulated in U87MG cells upon BRV treatment*. Differentiating miRNAs are listed with their *p*-values obtained by paired t-test (*p*val). In the table are also indicated false discovery rate values (FDR), and folds of deregulation expressed in logarithmic scale (log2 fold). (PPTX 64 kb)

Additional file 7: Figure S7. *Ectopic expression of miR-195-5p induces accumulation of cells in G0/G1*. a-b) Proliferation assay (a) and viability assay (b) in U87MG cells transfected with miR-107 mimic or control. Cells were collected and counted at the indicated time points. c) Typical experiment. U87MG were transfected with control (left) or with mimic miR-195-5p mimic (right) and cultured for the following 48 h. Cell were then harvested, fixed in 80% ethanol, stained with PI and analysed by flow cytometry for DNA content (see methods). d) U87MG cells morphology upon miR-195-5p exogenous expression. e) qRT-PCR of miR-195-5p in U87MG cells depleted for miR-195-5p (inh miR-195-5p) and treated with BRV or LCM (IC20). (PPTX 3351 kb)

Additional file 8: Figure S8. a) Transwell migration assay in U87MG cells upon miR-195-5p exogenous expression. b) qRT-PCR of miR-107 in U87MG cells depleted for miR-107 (inh miR-107) and treated with BRV or LCM (IC20). (PPTX 44 kb)

## Abbreviations

AED: Anti-epileptic drugs
BRV: Brivaracetam
EIAED: Enzyme-inducing anticonvulsant drugs
GBM: Glioblastoma
HUVECs: Human umbilical vein endothelial cells
LCM: Lacosamide
LEV: Levetiracetam
miRNA: microRNA
MRPs: Multidrug resistance proteins
OS: Overall survival
PFS: Progression-free survival
TMZ: Temozolomide
VPA: Valproic acid
