# Supplementary figures and images for: In vitro antineoplastic effects of brivaracetam and lacosamide on human glioma cells
Source: J Exp Clin Cancer Res. 2017 Jun 6;36:76. doi: 10.1186/s13046-017-0546-9 (PMC5460451; doi:10.1186/s13046-017-0546-9)

## Slide 1
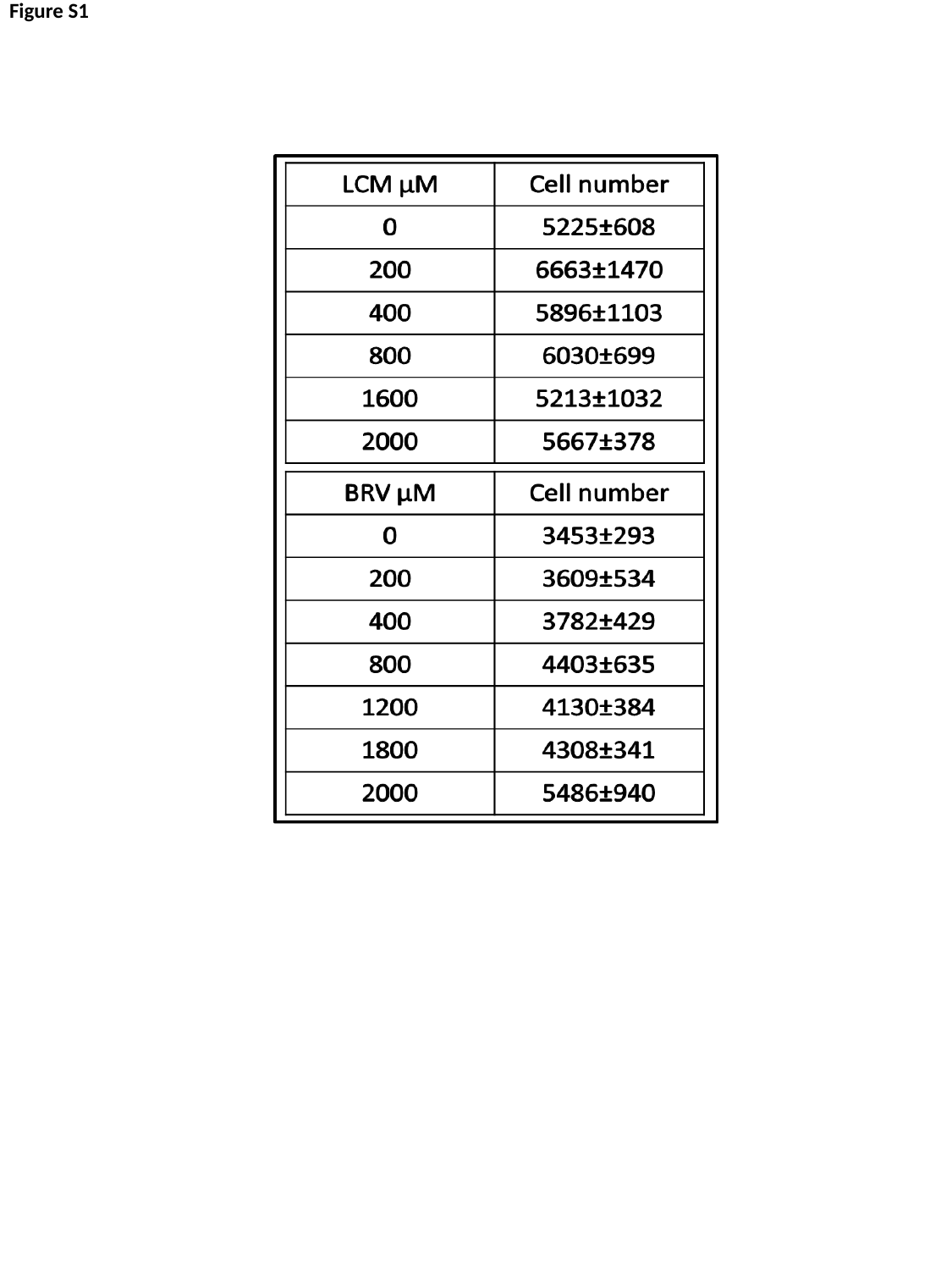

Figure S1

Supplement: Supplementary file 1 — Brivaracetam and lacosamide treatments displayed no cytotoxic effect on normal human fibroblast exposed to increasing drugs concentration. Data refers to at least three independent experiments and are expressed as cell number ± SD. (PPTX 106 kb) [file 13046_2017_546_MOESM1_ESM.pptx]

## Slide 1
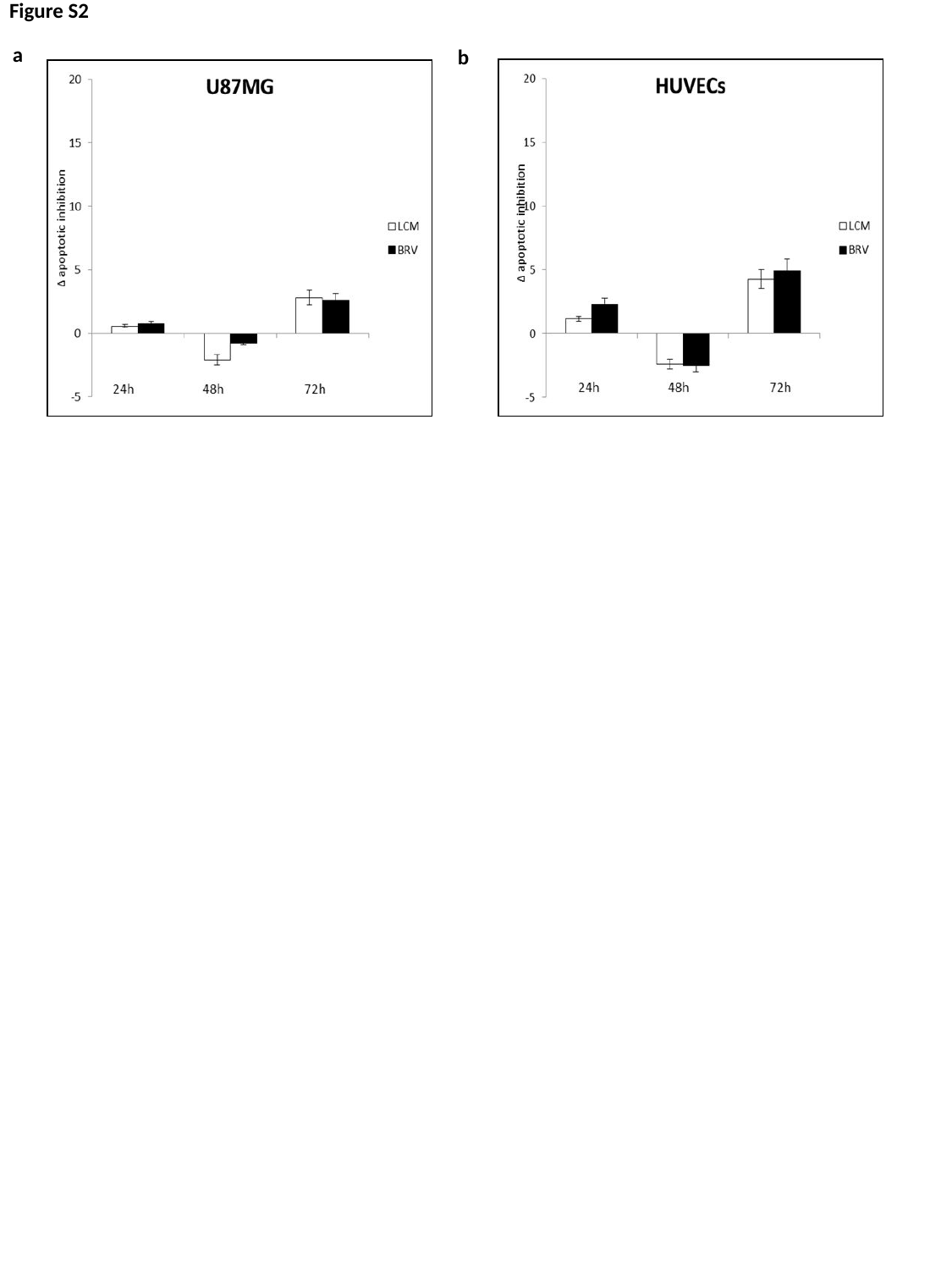

Figure S2
a
b

Supplement: Supplementary file 2 — a-b) Apoptosis analysis studied on the cell line U87MG (a) and on HUVECs (b) after treatment with BRV (IC20, grey histogram) and LCM (IC20, empty histogram) at different time points 24, 48 and 72 h. Data are expressed as difference in the % of apoptotic cells between treated and untreated cells (Δ apoptotic inhibition). Data refer to at least three independent experiments, error bars represent the SD. (PPTX 72 kb) [file 13046_2017_546_MOESM2_ESM.pptx]

## Slide 1
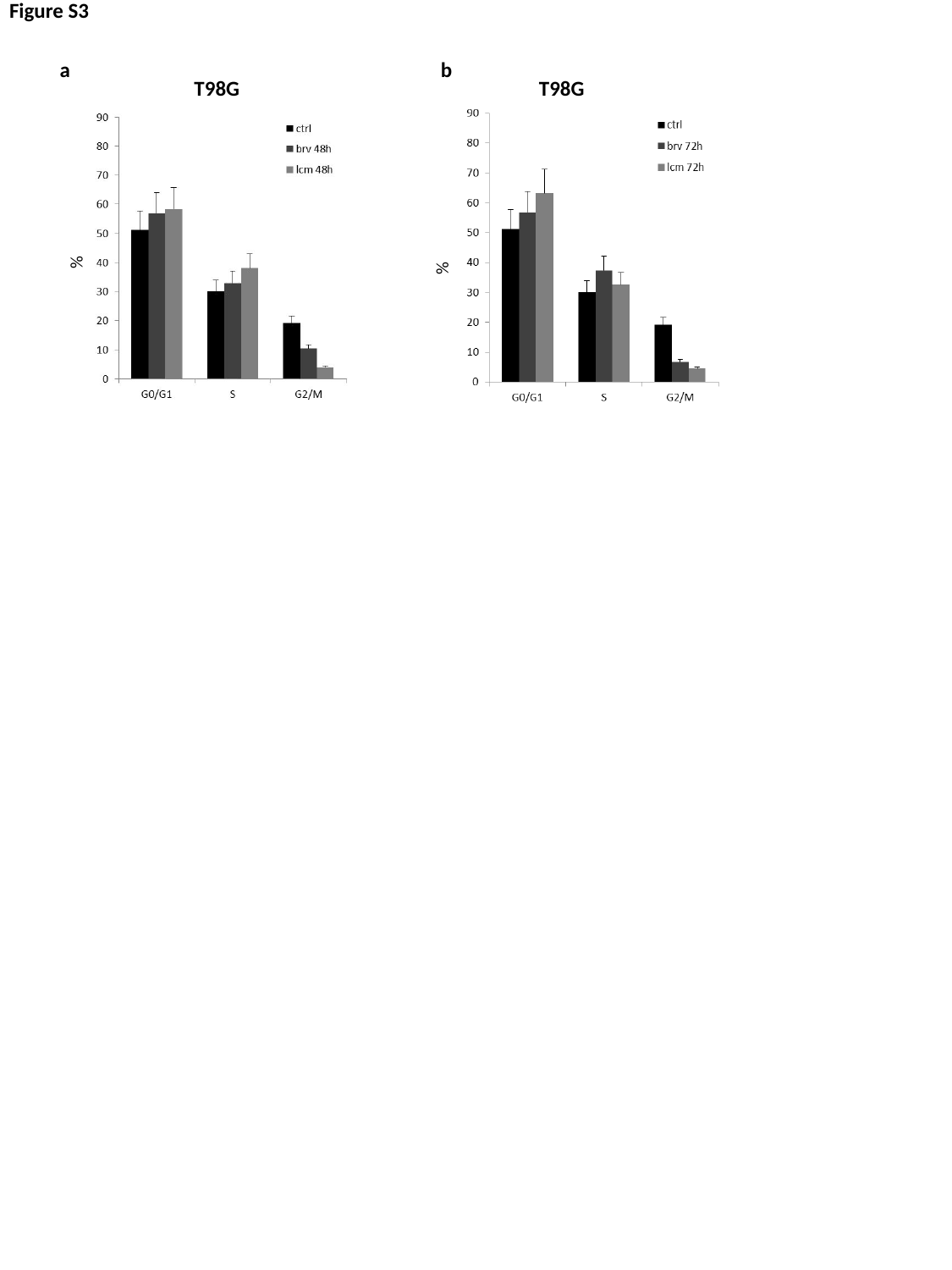

Figure S3
a
b
T98G
T98G
%
%

Supplement: Supplementary file 3 — Brivaracetam and lacosamide treatments induces accumulation of cells in G0/G1. a-b) Distribution of T98G cells in the different phases of the cell cycle upon 48 h (a) or 72 h (b) BRV or LCM treatments (IC20). Data are expressed as percentage of cell in a specific phase (G0/G1, S, G2/M) and refers to at least four independent experiments. Statistical evaluation was performed by the student’s t-test. Histogram bars represent mean ± standard deviation of at least three independent replicates. (PPTX 65 kb) [file 13046_2017_546_MOESM3_ESM.pptx]

## Slide 1
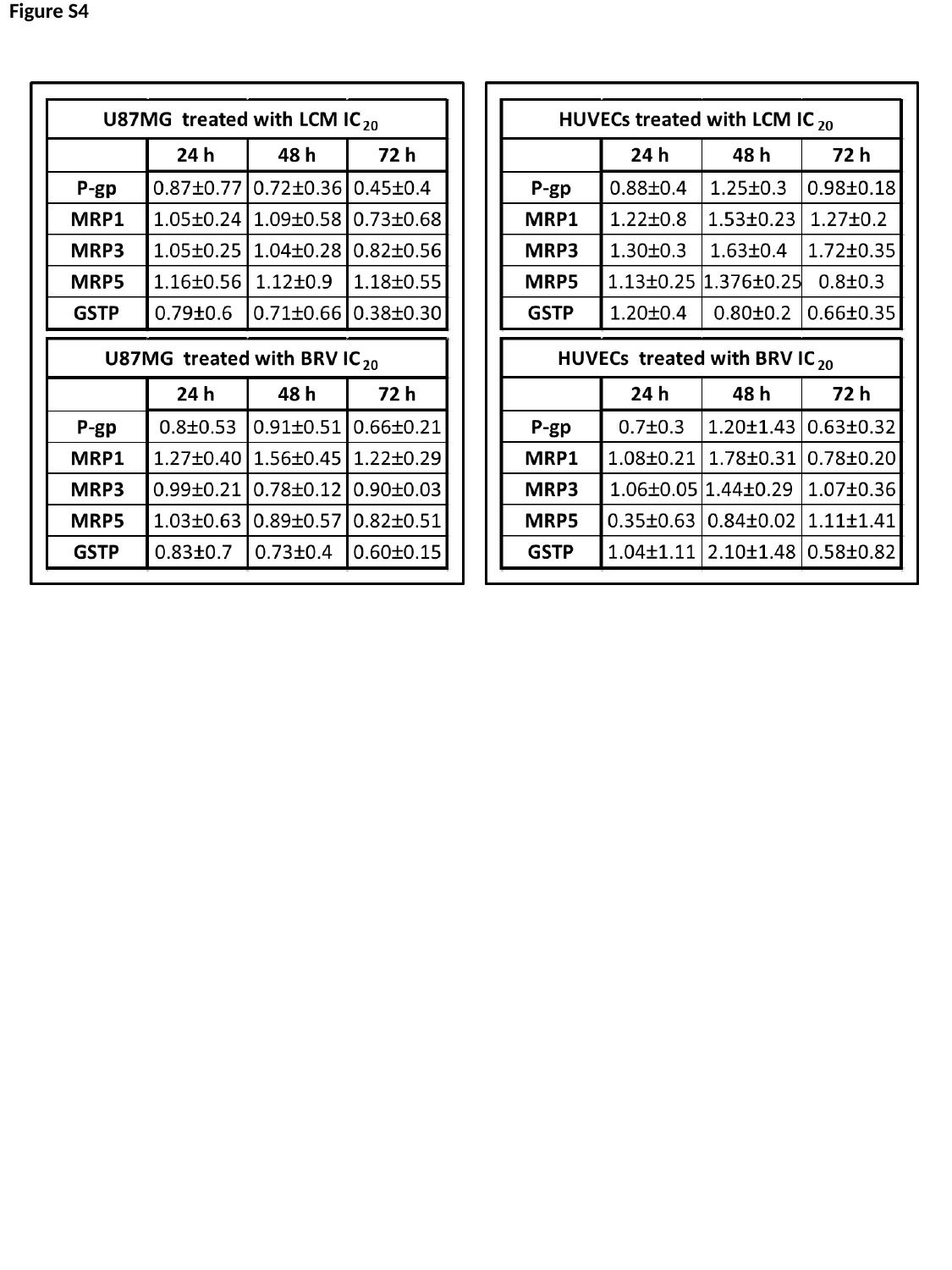

Figure S4

Supplement: Supplementary file 4 — Brivaracetam and lacosamide treatments have no impact on chemoresistence induction. Drug resistance molecules expression and modulation in U87MG cell line as detected by flow cytometry. In vitro cultured cells were treated with an IC20 concentration of BRV (grey histogram) or LCM (empty histogram) for 72 h, harvested and labelled with the specific antibodies (see methods). Data are expressed as fold increase/decrease (means ± SD) of treated cells compared to basal expression (averages of mean fluorescence intensity of treated cells/averages of mean fluorescence intensity of untreated cells). (PPTX 196 kb) [file 13046_2017_546_MOESM4_ESM.pptx]

## Slide 1
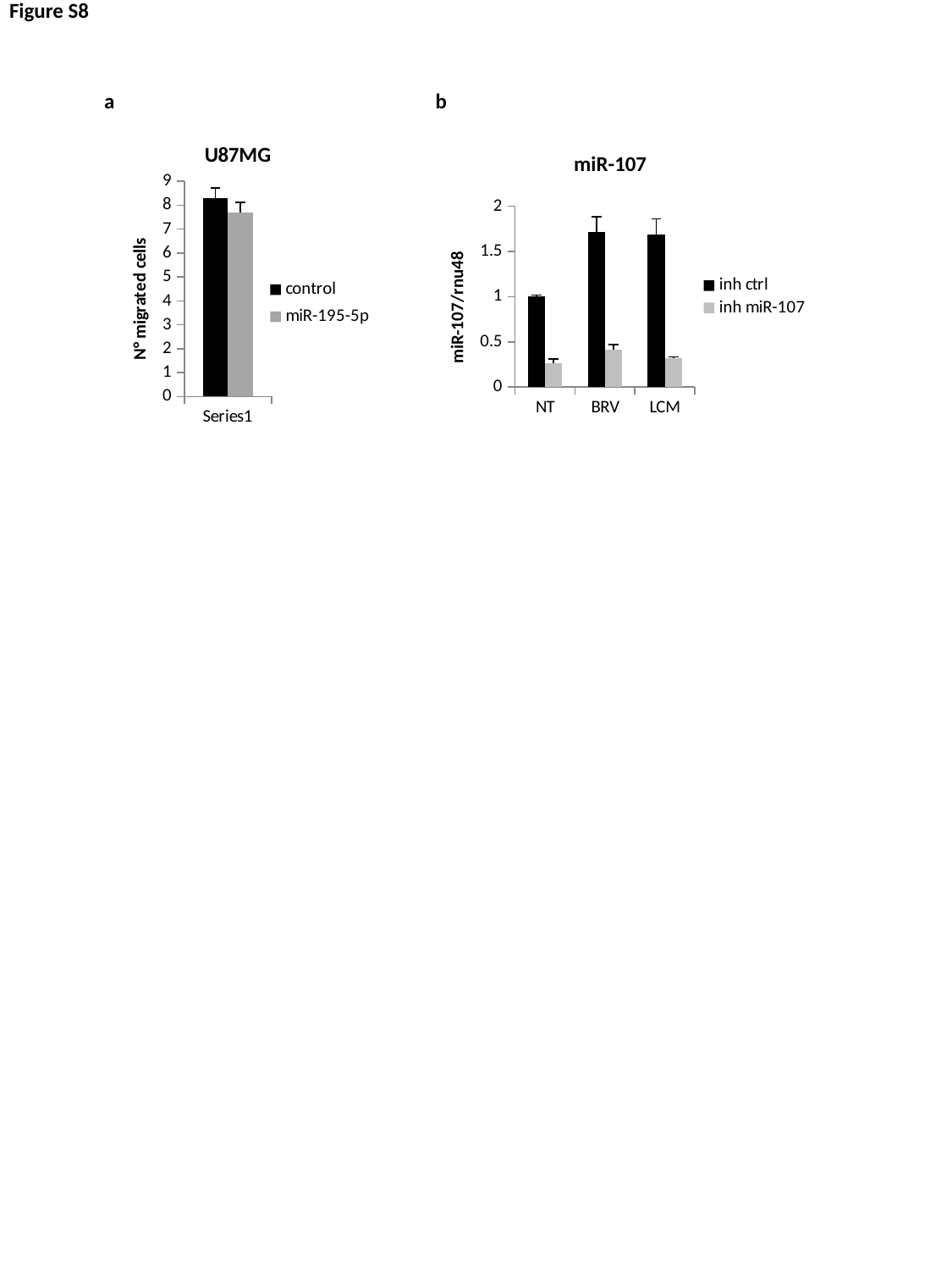

Figure S8
a
b
[unsupported chart]
### Chart: miR-107
| Category | | |
|---|---|---|
| NT | 1.000092232152296 | 0.267713495299204 |
| BRV | 1.714226943612514 | 0.411614659317373 |
| LCM | 1.688249440606755 | 0.32063530556327 |

Supplement: Supplementary file 8 — a) Transwell migration assay in U87MG cells upon miR-195-5p exogenous expression. b) qRT-PCR of miR-107 in U87MG cells depleted for miR-107 (inh miR-107) and treated with BRV or LCM (IC20). (PPTX 44 kb) [file 13046_2017_546_MOESM8_ESM.pptx]
