# Supplementary material for: In vitro antineoplastic effects of brivaracetam and lacosamide on human glioma cells
Source: J Exp Clin Cancer Res. 2017 Jun 6;36:76. doi: 10.1186/s13046-017-0546-9 (PMC5460451; doi:10.1186/s13046-017-0546-9)
Supplement: Supplementary file 5 — miRNAs modulated in U87MG cells upon LCM treatment. Differentiating miRNAs are listed with their p-values obtained by paired t-test (pval). In the table are also indicated false discovery rate values (FDR), and folds of deregulation expressed in logarithmic scale (log2 fold). (PPTX 63 kb) [file 13046_2017_546_MOESM5_ESM.pptx]

## Slide 1
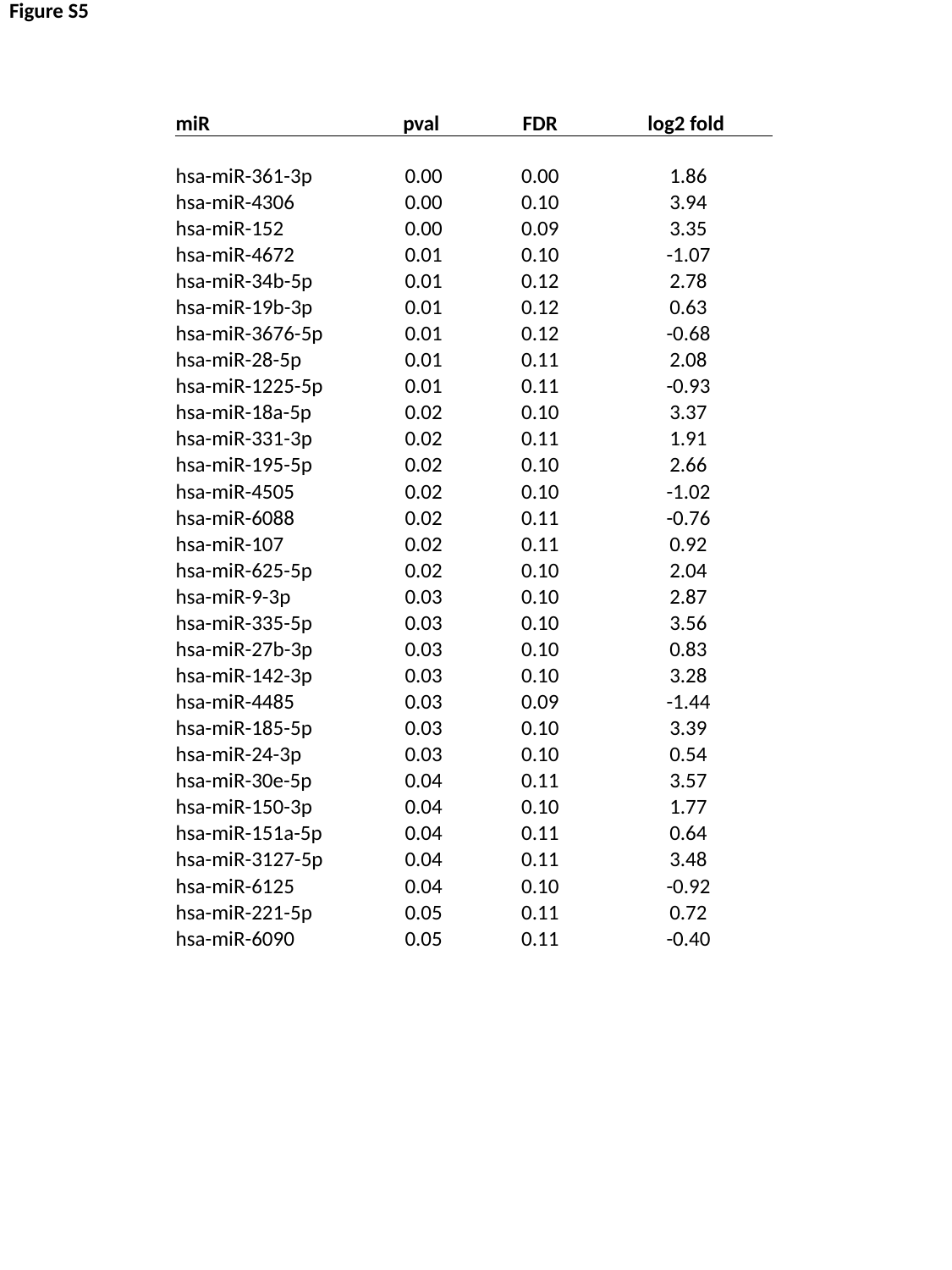

Figure S5
| miR | pval | FDR | log2 fold |
| --- | --- | --- | --- |
| | | | |
| hsa-miR-361-3p | 0.00 | 0.00 | 1.86 |
| hsa-miR-4306 | 0.00 | 0.10 | 3.94 |
| hsa-miR-152 | 0.00 | 0.09 | 3.35 |
| hsa-miR-4672 | 0.01 | 0.10 | -1.07 |
| hsa-miR-34b-5p | 0.01 | 0.12 | 2.78 |
| hsa-miR-19b-3p | 0.01 | 0.12 | 0.63 |
| hsa-miR-3676-5p | 0.01 | 0.12 | -0.68 |
| hsa-miR-28-5p | 0.01 | 0.11 | 2.08 |
| hsa-miR-1225-5p | 0.01 | 0.11 | -0.93 |
| hsa-miR-18a-5p | 0.02 | 0.10 | 3.37 |
| hsa-miR-331-3p | 0.02 | 0.11 | 1.91 |
| hsa-miR-195-5p | 0.02 | 0.10 | 2.66 |
| hsa-miR-4505 | 0.02 | 0.10 | -1.02 |
| hsa-miR-6088 | 0.02 | 0.11 | -0.76 |
| hsa-miR-107 | 0.02 | 0.11 | 0.92 |
| hsa-miR-625-5p | 0.02 | 0.10 | 2.04 |
| hsa-miR-9-3p | 0.03 | 0.10 | 2.87 |
| hsa-miR-335-5p | 0.03 | 0.10 | 3.56 |
| hsa-miR-27b-3p | 0.03 | 0.10 | 0.83 |
| hsa-miR-142-3p | 0.03 | 0.10 | 3.28 |
| hsa-miR-4485 | 0.03 | 0.09 | -1.44 |
| hsa-miR-185-5p | 0.03 | 0.10 | 3.39 |
| hsa-miR-24-3p | 0.03 | 0.10 | 0.54 |
| hsa-miR-30e-5p | 0.04 | 0.11 | 3.57 |
| hsa-miR-150-3p | 0.04 | 0.10 | 1.77 |
| hsa-miR-151a-5p | 0.04 | 0.11 | 0.64 |
| hsa-miR-3127-5p | 0.04 | 0.11 | 3.48 |
| hsa-miR-6125 | 0.04 | 0.10 | -0.92 |
| hsa-miR-221-5p | 0.05 | 0.11 | 0.72 |
| hsa-miR-6090 | 0.05 | 0.11 | -0.40 |
