# Supplementary material for: In vitro antineoplastic effects of brivaracetam and lacosamide on human glioma cells
Source: J Exp Clin Cancer Res. 2017 Jun 6;36:76. doi: 10.1186/s13046-017-0546-9 (PMC5460451; doi:10.1186/s13046-017-0546-9)
Supplement: Supplementary file 6 — miRNAs modulated in U87MG cells upon BRV treatment. Differentiating miRNAs are listed with their p-values obtained by paired t-test (pval). In the table are also indicated false discovery rate values (FDR), and folds of deregulation expressed in logarithmic scale (log2 fold). (PPTX 64 kb) [file 13046_2017_546_MOESM6_ESM.pptx]

## Slide 1
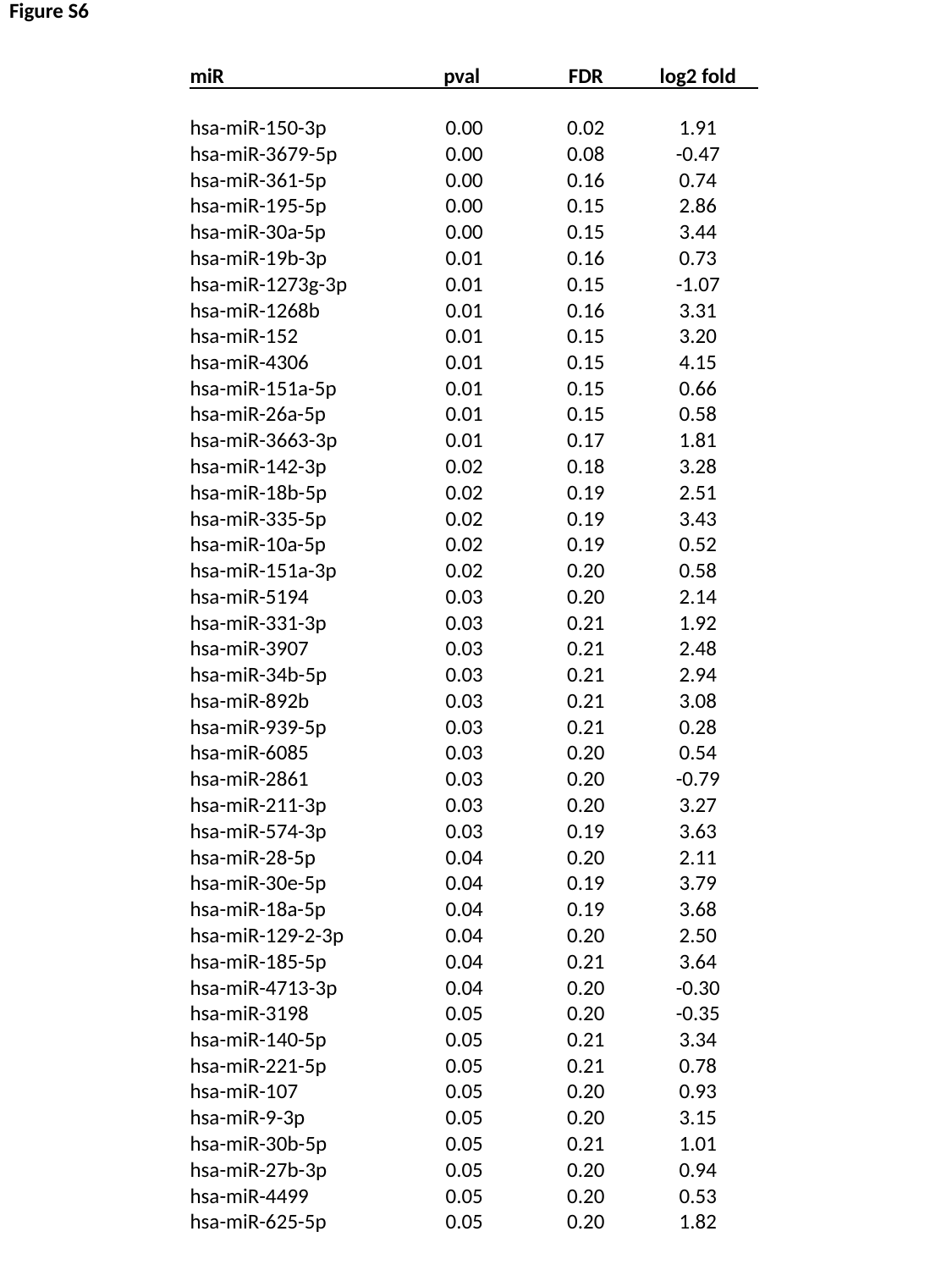

Figure S6
| miR | pval | FDR | log2 fold |
| --- | --- | --- | --- |
| | | | |
| hsa-miR-150-3p | 0.00 | 0.02 | 1.91 |
| hsa-miR-3679-5p | 0.00 | 0.08 | -0.47 |
| hsa-miR-361-5p | 0.00 | 0.16 | 0.74 |
| hsa-miR-195-5p | 0.00 | 0.15 | 2.86 |
| hsa-miR-30a-5p | 0.00 | 0.15 | 3.44 |
| hsa-miR-19b-3p | 0.01 | 0.16 | 0.73 |
| hsa-miR-1273g-3p | 0.01 | 0.15 | -1.07 |
| hsa-miR-1268b | 0.01 | 0.16 | 3.31 |
| hsa-miR-152 | 0.01 | 0.15 | 3.20 |
| hsa-miR-4306 | 0.01 | 0.15 | 4.15 |
| hsa-miR-151a-5p | 0.01 | 0.15 | 0.66 |
| hsa-miR-26a-5p | 0.01 | 0.15 | 0.58 |
| hsa-miR-3663-3p | 0.01 | 0.17 | 1.81 |
| hsa-miR-142-3p | 0.02 | 0.18 | 3.28 |
| hsa-miR-18b-5p | 0.02 | 0.19 | 2.51 |
| hsa-miR-335-5p | 0.02 | 0.19 | 3.43 |
| hsa-miR-10a-5p | 0.02 | 0.19 | 0.52 |
| hsa-miR-151a-3p | 0.02 | 0.20 | 0.58 |
| hsa-miR-5194 | 0.03 | 0.20 | 2.14 |
| hsa-miR-331-3p | 0.03 | 0.21 | 1.92 |
| hsa-miR-3907 | 0.03 | 0.21 | 2.48 |
| hsa-miR-34b-5p | 0.03 | 0.21 | 2.94 |
| hsa-miR-892b | 0.03 | 0.21 | 3.08 |
| hsa-miR-939-5p | 0.03 | 0.21 | 0.28 |
| hsa-miR-6085 | 0.03 | 0.20 | 0.54 |
| hsa-miR-2861 | 0.03 | 0.20 | -0.79 |
| hsa-miR-211-3p | 0.03 | 0.20 | 3.27 |
| hsa-miR-574-3p | 0.03 | 0.19 | 3.63 |
| hsa-miR-28-5p | 0.04 | 0.20 | 2.11 |
| hsa-miR-30e-5p | 0.04 | 0.19 | 3.79 |
| hsa-miR-18a-5p | 0.04 | 0.19 | 3.68 |
| hsa-miR-129-2-3p | 0.04 | 0.20 | 2.50 |
| hsa-miR-185-5p | 0.04 | 0.21 | 3.64 |
| hsa-miR-4713-3p | 0.04 | 0.20 | -0.30 |
| hsa-miR-3198 | 0.05 | 0.20 | -0.35 |
| hsa-miR-140-5p | 0.05 | 0.21 | 3.34 |
| hsa-miR-221-5p | 0.05 | 0.21 | 0.78 |
| hsa-miR-107 | 0.05 | 0.20 | 0.93 |
| hsa-miR-9-3p | 0.05 | 0.20 | 3.15 |
| hsa-miR-30b-5p | 0.05 | 0.21 | 1.01 |
| hsa-miR-27b-3p | 0.05 | 0.20 | 0.94 |
| hsa-miR-4499 | 0.05 | 0.20 | 0.53 |
| hsa-miR-625-5p | 0.05 | 0.20 | 1.82 |
