# Supplementary material for: In vitro antineoplastic effects of brivaracetam and lacosamide on human glioma cells
Source: J Exp Clin Cancer Res. 2017 Jun 6;36:76. doi: 10.1186/s13046-017-0546-9 (PMC5460451; doi:10.1186/s13046-017-0546-9)
Supplement: Supplementary file 7 — Ectopic expression of miR-195-5p induces accumulation of cells in G0/G1. a-b) Proliferation assay (a) and viability assay (b) in U87MG cells transfected with miR-107 mimic or control. Cells were collected and counted at the indicated time points. c) Typical experiment. U87MG were transfected with control (left) or with mimic miR-195-5p mimic (right) and cultured for the following 48 h. Cell were then harvested, fixed in 80% ethanol, stained with PI and analysed by flow cytometry for DNA content (see methods). d) U87MG cells morphology upon miR-195-5p exogenous expression. e) qRT-PCR of miR-195-5p in U87MG cells depleted for miR-195-5p (inh miR-195-5p) and treated with BRV or LCM (IC20). (PPTX 3351 kb) [file 13046_2017_546_MOESM7_ESM.pptx]

## Slide 1
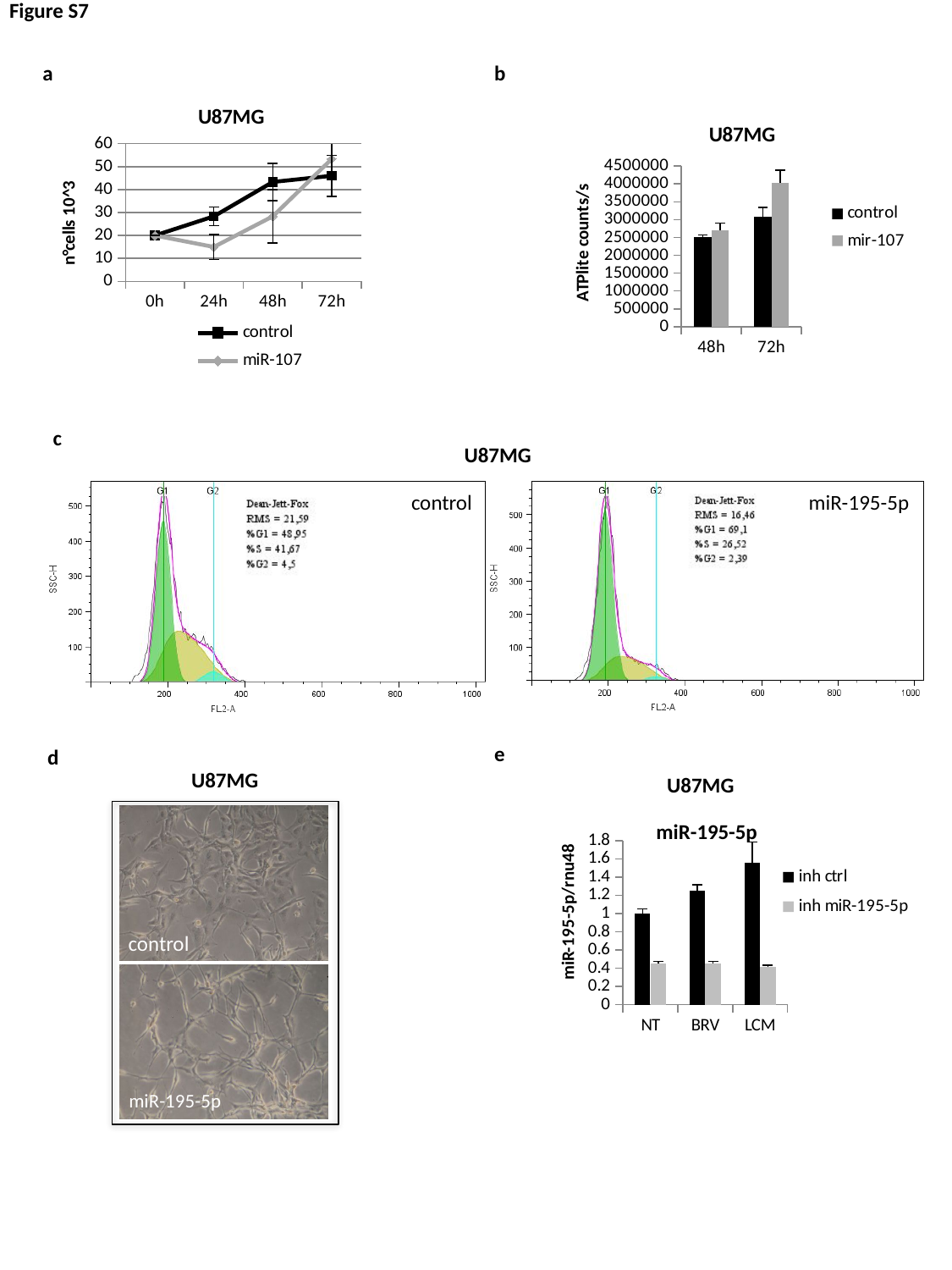

Figure S7
a
### Chart: U87MG
| Category | | |
|---|---|---|
| 0h | 20.0 | 20.0 |
| 24h | 28.33333333333328 | 15.0 |
| 48h | 43.33333333333334 | 28.33333333333328 |
| 72h | 46.0 | 53.33333333333334 |b
### Chart: U87MG
| Category | | |
|---|---|---|
| 48h | 2515501.0 | 2710025.0 |
| 72h | 3072024.0 | 4020869.0 |c
U87MG
control
miR-195-5p
e
U87MG
### Chart: miR-195-5p
| Category | | |
|---|---|---|
| NT | 1.000661926967791 | 0.448361766745732 |
| BRV | 1.248640553147031 | 0.450881426598942 |
| LCM | 1.553433580731613 | 0.419043865788537 |d
U87MG
control
miR-195-5p
